# Estimation of the global prevalence and burden of insomnia: a systematic literature review-based analysis

**DOI:** 10.1016/j.smrv.2025.102121

**Published:** 2025-06-25

**Authors:** Adam V. Benjafield, Fatima H Sert Kuniyoshi, Atul Malhotra, Jennifer L. Martin, Charles M. Morin, Leonie F. Maurer, Peter A. Cistulli, Jean-Louis Pépin, Emerson M. Wickwire

**Affiliations:** a ResMed Science Center, Sydney, NSW, Australia; b ResMed Science Center, San Diego, CA, USA; c University California San Diego, La Jolla, CA, USA; d Geriatric Research, Education and Clinical Center, VA Greater Los Angeles, Los Angeles, CA, USA; e David Geffen School of Medicine, University of California, Los Angeles, Canada; f Université Laval, Québec City, Canada; g Mementor DE GmbH, Leipzig, Germany; h Charles Perkins Centre, Faculty of Medicine and Health, University Sydney, and Department of Respiratory and Sleep Medicine, Royal North Shore Hospital, Sydney, NSW, Australia; i HP2 Laboratory, Inserm U1300, Grenoble Alps University, Grenoble, France; j University of Maryland School of Medicine, Baltimore, MD, USA

**Keywords:** Insomnia, Prevalence, Sleep, Global

## Abstract

Insomnia is common, is associated with major adverse medical and mental health outcomes, has a negative impact on quality of life, and has significant economic consequences. However, little is known about the global insomnia burden. This systematic review estimated the global prevalence of insomnia in adults. PubMed and Embase were searched (terms “insomnia,” “prevalence,” and “general population”) to identify relevant peer-reviewed studies (final search 2–3 Sep 2024). Included studies had the highest data quality and lowest risk of bias, and reported clinically relevant insomnia prevalence in the general population. Insomnia prevalence estimates were applied to United Nations (UN) population data using a country-specific study (if available) or the highest-quality study (if no country-specific study). Of 1651 potential records, 18 studies (262,582 participants) were included. Thirty-one of 237 UN/World Bank-recognized countries/territories had a suitable nation-specific adult insomnia prevalence estimate. 852,325,091 adults (95 % confidence interval 830,354,161–874,309,252) were estimated to have insomnia (global prevalence: 16.2 %) and 414,967,941 were estimated to have severe insomnia (7.9 %). Insomnia and severe insomnia were more prevalent in females versus males across all age groups. The high global prevalence of insomnia disorder reinforces the need for comprehensive public health and clinical sleep health initiatives worldwide.

**Registration:**

PROSPERO: CRD42024581410.

## 1. Introduction

Insomnia disorder, defined as frequent and persistent difficulty initiating and/or maintaining sleep with associated daytime consequences [1,2], is among the most common sleep disorders in adults worldwide [3–7]. Although once considered secondary to medical and psychiatric issues, insomnia is now widely recognized as an independent condition that warrants diagnosis and treatment [1,2,6].

Insomnia is associated with a broad range of adverse medical (e.g., cardiovascular [8], metabolic [9], and neurodegenerative [10] diseases) and mental health (e.g., depression [11,12], anxiety [12], chronic pain [13], substance misuse [12,14]) outcomes, and diminished quality of life [15,16]. In addition, insomnia is associated with a dramatic increase in the economic costs borne by multiple stakeholders, including payers (increased costs and healthcare resource utilization), employers (diminished workplace productivity and increased accident risk [17, 18]), and society in general [19].

In other areas of medicine, understanding the global burden of disease is recognized as a key scientific priority to guide prevention, treatment, and policy initiatives, and to help ensure appropriate allocation of scare resources [20–22]. A prerequisite to understanding disease burden is to estimate the population prevalence of a condition. However, despite the globally recognized burden of insomnia and the availability of evidence-based treatments, little is known regarding the actual number of people with insomnia from a global health perspective. To address these important knowledge gaps, this study undertook a systematic literature review then performed a quantitative synthesis of available data to estimate the global prevalence of insomnia in adults.

## 2. Methods

### 2.1. Overview

The systematic literature review was registered with PROSPERO (CRD42024581410), and a detailed protocol can be found at that listing. Our approach was to apply published nation-specific estimates of the population prevalence of insomnia in adults (age ≥20 years) to current population estimates for all countries worldwide. Insomnia prevalence estimates were obtained via literature search as described below. Current global population data were obtained from the United Nations (UN) World Population Prospects 2022 [23]. Conservative approaches were applied throughout. Ethics oversight was not required as the data used in this analysis were anonymized and publicly available.

### 2.2. Inclusion criteria

To be included in this review, empirical studies were required to estimate the prevalence of clinically significant insomnia (i.e., including metrics of both sleep and daytime function, as defined below) within the general population. Additional inclusion criteria included peer-reviewed scientific publication in English language. Conference abstracts or proceedings, modeling studies based on previously published data, and reviews were excluded. Studies based on clinical samples (rather than the overall population) were also excluded.

### 2.3. Insomnia definition

Our primary outcome was clinically significant insomnia. Since reference studies were published over multiple decades, we expected high heterogeneity in operational definitions of insomnia across studies. Therefore, we elected to define clinically significant insomnia based on both nighttime and daytime symptoms of insomnia, including difficulty initiating sleep, difficulty maintaining sleep, and/or nonrestorative sleep, with associated daytime consequence(s). For nations without a country-specific estimate of insomnia prevalence, definitions of “insomnia” and “severe insomnia” were based on the definitions used in highest-quality reference study available. In that study [23], insomnia was defined as chronic (endorsing one DSM-IV sleep-related insomnia symptom, with daytime consequence) or severe (endorsing two or more DSM-IV insomnia symptoms, with daytime consequence).

### 2.4. Population estimates

The UN World Population Prospects 2022 [23] data are based on >1700 national population censuses since 1950 and other data sources. The document provides nation-specific population estimates for 237 nations or areas worldwide, and these estimates are stratified by biological sex and age in five-year increments.

### 2.5. Information sources

The PubMed and Embase databases plus the reference lists of identified studies were searched for this study.

### 2.6. Data searches

The following search terms were used: “insomnia,” “prevalence,” and “population.” No restriction of timeframe was applied. A preliminary literature search was done in 2023 and the final literature search reported here was performed September 2–3, 2024.

### 2.7. Study selection

To determine which insomnia prevalence estimates to apply to UN population data, we applied four criteria: 1) for studies that reported both age and sex together (i.e., prevalence of insomnia in males and females, separately, by age), stratified results were used; 2) for studies that reported insomnia prevalence by age and sex separately, age-based results were used (because age enabled more granular breakdown than sex); 3) for studies that reported insomnia prevalence by sex but not age, sex-based results were used; 4) for studies that reported only a single prevalence estimate, that estimate was applied to the entire population of that country. For countries with >1 potential reference study, expert opinion was used to select the most methodologically rigorous study based on the criteria listed above. For countries for which no reference study was identified, we applied prevalence data from a large, well-conducted study on insomnia prevalence in adults, which included prevalence estimates based on biological sex and age categories [24].

### 2.8. Data collection process and data items

Data were extracted from reports and reviewed for accuracy by two independent reviewers (AVB and FHSK), with subsequent review by a third reviewer (EMW). The information extracted included study population, operational definitions of insomnia, and reported prevalence results.

### 2.9. Quality and risk of bias assessment

The quality of the studies was assessed using the Newcastle-Ottawa quality assessment Scale, tailored specifically for cross-sectional data and cohort studies [25]. Both study-level and outcome-level characteristics were evaluated to ensure the validity and reliability of the prevalence estimates. This process included documentation and evaluation of the following: type of study, sampling method, setting, and data collection period; sample size, demographics, and geographic representativeness; methods used for determining the presence of insomnia; definition of insomnia; subgroup analyses (e.g., prevalence by age groups, sex); and validity and reliability of the instruments used to measure insomnia.

### 2.10. Quantitative data synthesis

Using the approach above, we computed the nation-specific prevalence of insomnia in adults. Confidence interval (CI) values for the global estimate incorporate the sampling variability in insomnia prevalence estimates from the reference studies, but do not address potential biases in applying these estimates. The 95 % CI values for global insomnia prevalence estimates were calculated using the formula for the variance of a binomial random variable. Specifically, the global estimate can be written in the following way:

$$X=\sum_{i=1}^{K}N_{i}p_{i}$$

where $N_{i}$ is the population size of the ith subgroup (each subgroup is a sex-age group in a specific country) and $p_{i}$ is the insomnia prevalence estimate applied to the ith subgroup.

Given that several prevalence estimates were applied to a number of subgroups, the estimate can be rewritten by summing the population sizes of all subgroups to which each estimate was applied:

$$X=\sum_{j=1}^{M}M_{j}p_{j}$$

where $M_{j}$ is the total population size to which the jth insomnia prevalence estimate was applied.

The insomnia prevalence estimate $p_{j}$ has variance $p_{j}\left(1-p_{j}\right)/n_{j}$, where $n_{j}$ is the number of subjects used to estimate $p_{j}$. The participants used in each prevalence estimate are independent, meaning that the variance of the global estimate is:

$$\mathrm{Var}(X)=\sum_{j=1}^{M}M_{j}^{2}\mathrm{Var}\left(p_{j}\right)=\sum_{j=1}^{M}M_{j}^{2}p_{j}\left(1-p_{j}\right)/n_{j}$$

and the 95 % CI is $X\pm 1.96\sqrt{\mathrm{Var}(X)}$.

To visualize the increasing burden of insomnia with advancing age, we also calculated and plotted the cumulative number of adults with insomnia disorder by sex across the lifespan (e.g., ≥20, ≥25, ≥30 years, etc.). These cumulative categories were derived from the sum of all individuals aged ≥20 years within each threshold, based on age- and sex-specific prevalence estimates applied to UN population data.

### 2.11. Sensitivity analyses

Given that our approach aggregated data from heterogeneous studies, we performed multiple sensitivity analyses to ensure robustness of results: 1) the prevalence of severe insomnia (rather than the overall prevalence of insomnia) from the study by Leger and colleagues [24] was applied to all countries/territories without a reference study; 2) the prevalence of insomnia and severe insomnia from the study by Leger and colleagues [24] was applied to all countries; and 3) the global prevalence of severe insomnia was determined.

## 3. Results

### 3.1. Study details

**Fig. 1.**
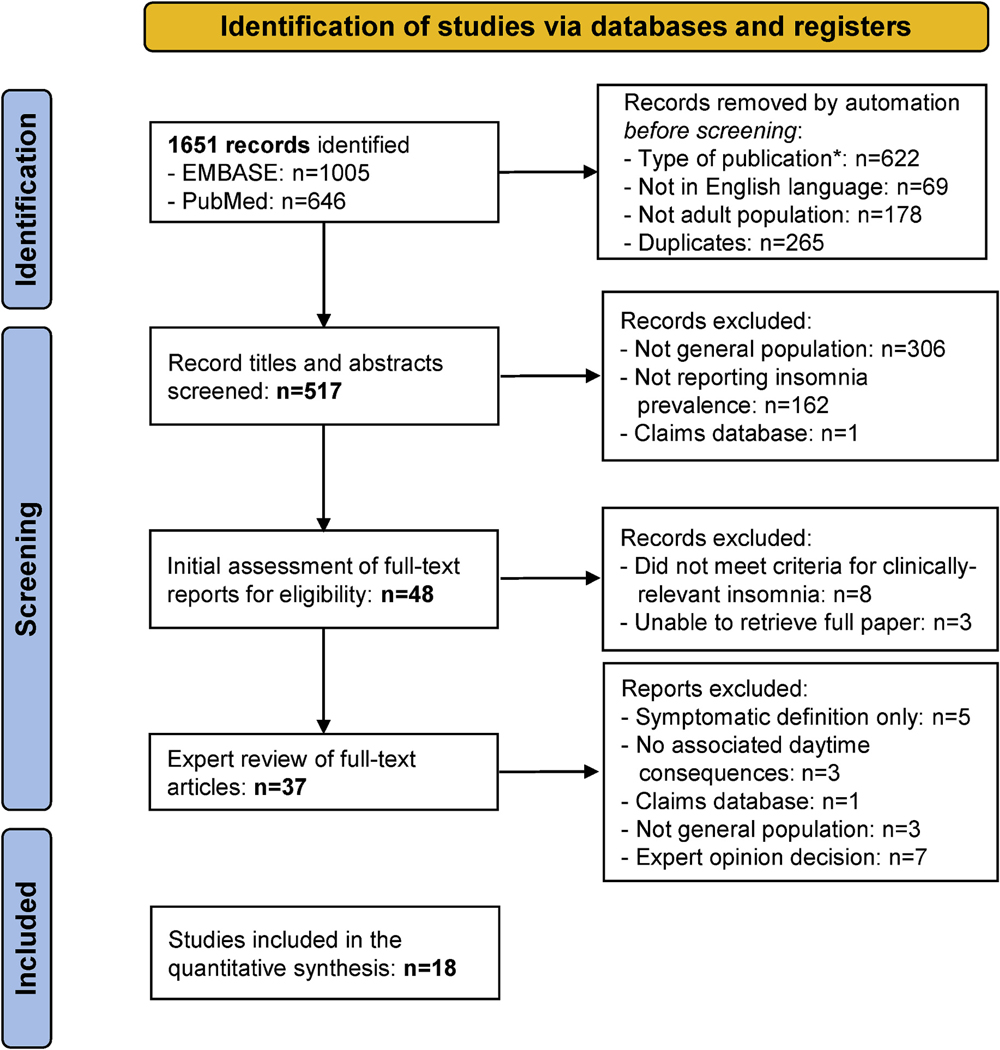
Flow chart of study selection.

A total of 1651 potential records were identified via databases and registers, of which 18 met the inclusion criteria and were therefore suitable for inclusion in the prevalence estimate calculations (Fig. 1); reasons for non-inclusion and the number of studies this issue was relevant for are shown in the figure. The included studies were chosen based on their quality and low risk of bias.

Of 237 countries/territories recognized by the United Nations/World Bank, 31 were found to have a suitable nation-specific prevalence estimate of insomnia in adults (Algeria, Andorra, Belgium, Brazil, Canada, China, France, French Polynesia, Germany, Greece, Guadeloupe, Italy, Japan, Mauritania, Morocco, New Caledonia, Norway, Malaysia, Mayotte, Qatar, Seychelles, South Korea, Spain, Sweden, Taiwan, The Netherlands, Tonga, Tunisia, Turkey, United Kingdom, United States of America) [4,5,24,26–40].

All included country-specific prevalence studies had a survey design (with data for China coming from a meta-analysis of studies reporting local insomnia prevalence data [28]). Sample sizes ranged from 1101 to 115,988, with a total of 262,582 participants across all 18 studies. Where participant sex was reported, there was an appropriate balance of males and females (the proportion of females ranged from 47.1 % to 54.5 %). Full details of all the utilized reference studies are displayed in Table 1. Assessment of the quality of the reference studies are shown in Table S1.

### 3.2. Insomnia definitions

Within reference studies (Table 1), insomnia was defined based on criteria from the International Classification of Sleep Disorders (ICSD) Second Edition (n = 4), ICSD Third Edition (n = 3), Diagnostic and Statistical Manual of Mental Disorders (DSM) Fourth Edition (IV) (n = 10), DSM Fifth Edition (n = 1), or the Insomnia Research Diagnostic Criteria (n = 1). One study included a validated measure of insomnia severity (Insomnia Self-Assessment Inventory). One other study included a meta-analysis of available studies which utilized the DSM-IV criteria (n = 1), validated measures of insomnia severity (Athens Insomnia Scale, n = 2; Pittsburgh Sleep Quality Index, n = 10), and studies using non-validated measures (n = 4).

### 3.3. Global prevalence of insomnia in adults

#### 3.3.1. Overall

**Fig. 2.**
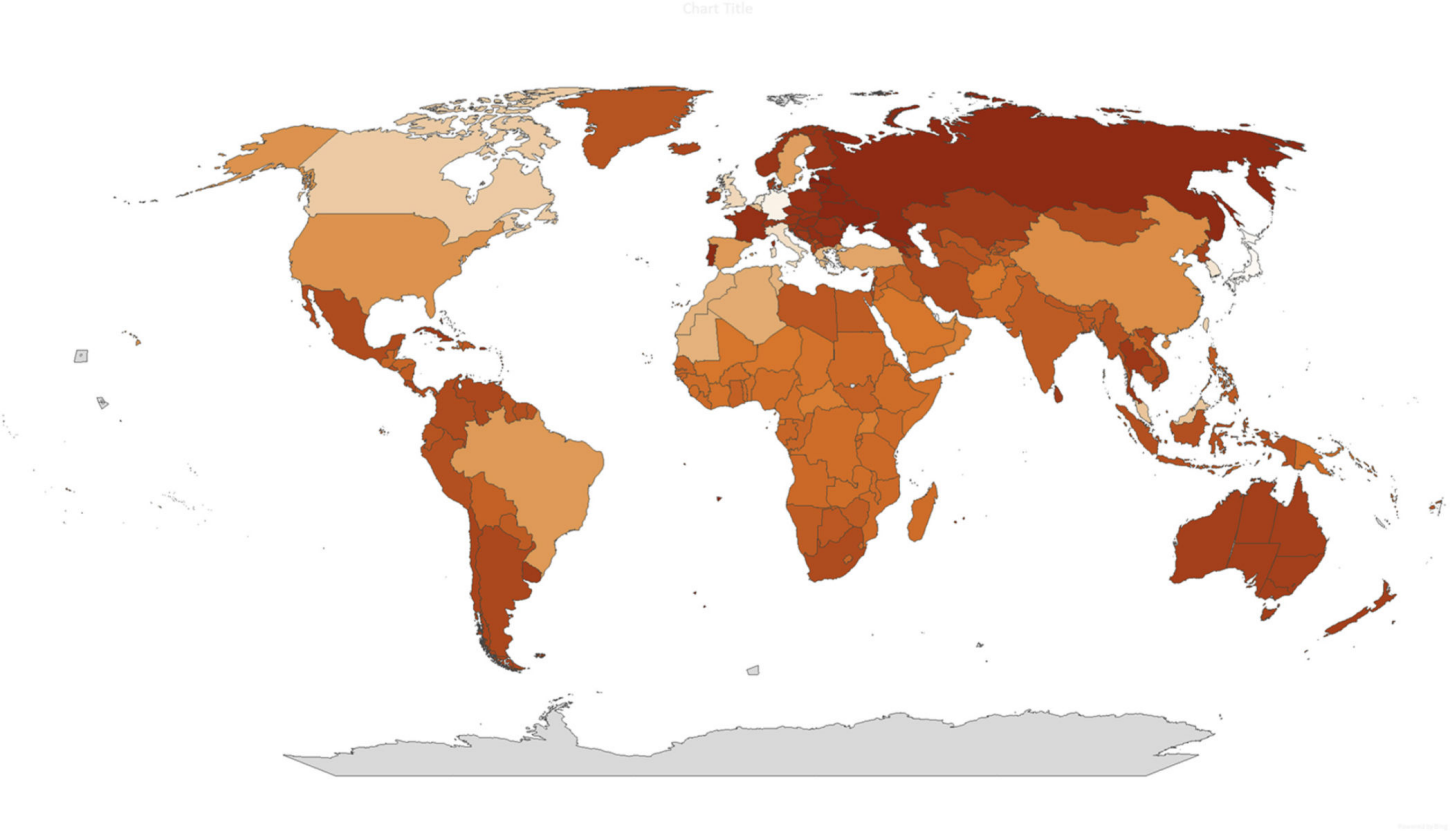
Global heat map for insomnia disorder in adults.

An estimated 852,325,091 adults (95 % CI 830,354,161; 874,309,252) met our definition of insomnia. This result translates to a global prevalence of chronic, clinically relevant insomnia in adults of 16.2 %. When expressed as a prevalence rate, the insomnia burden for each country is shown as a heat map (Fig. 2). The ten countries that had the highest estimated prevalence of insomnia disorder were Bangladesh, Brazil, China, India, Indonesia, Mexico, Nigeria, Pakistan, Russia, and United States of America (Fig. S1). A summary of the number of affected adults and the overall insomnia prevalence rate for each country, region or territory is provided alphabetically in Table S2.

#### 3.3.2. By age and biological sex

**Fig. 3.**
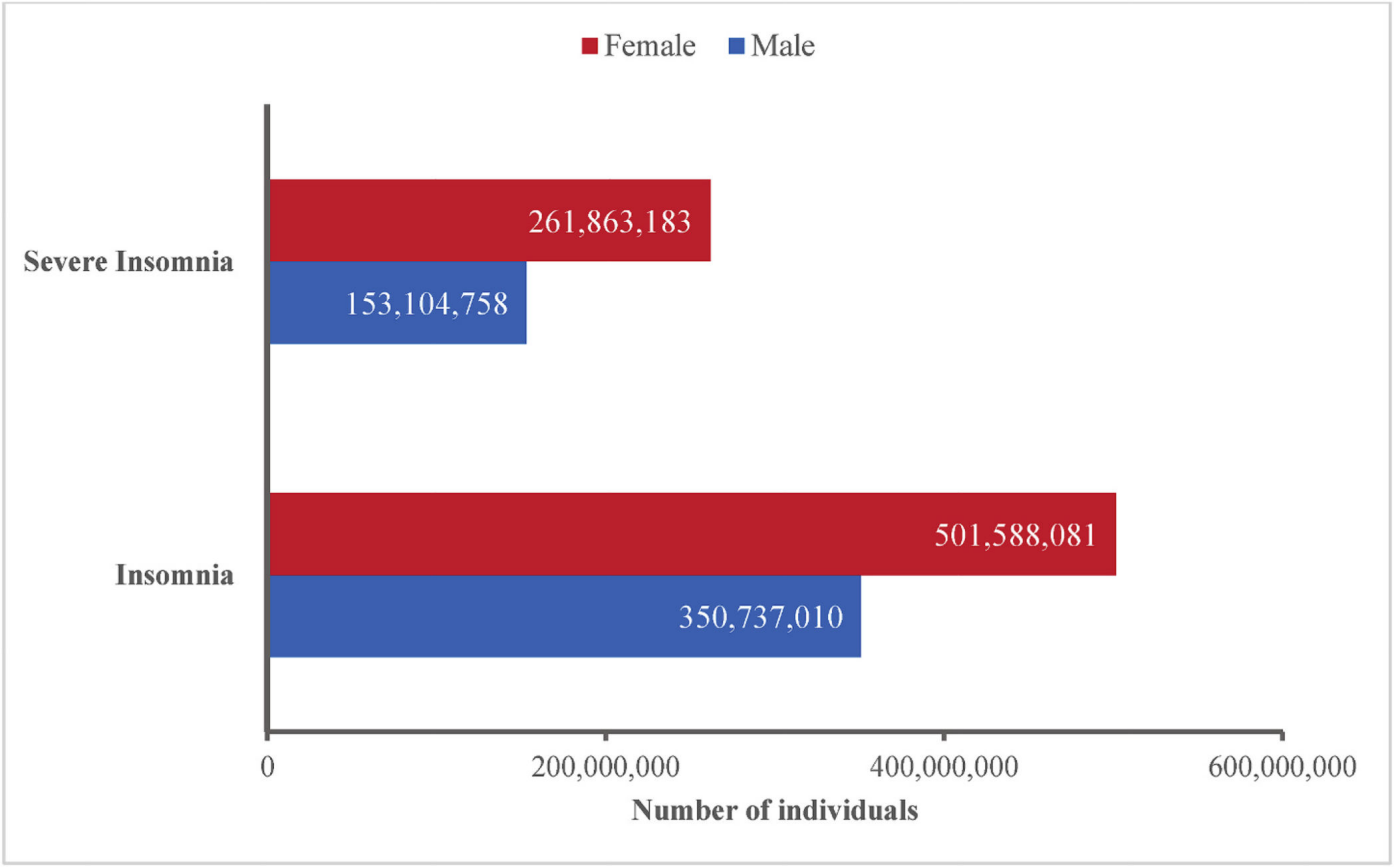
Number of adult males and females with insomnia disorder and severe insomnia.

**Fig. 4.**
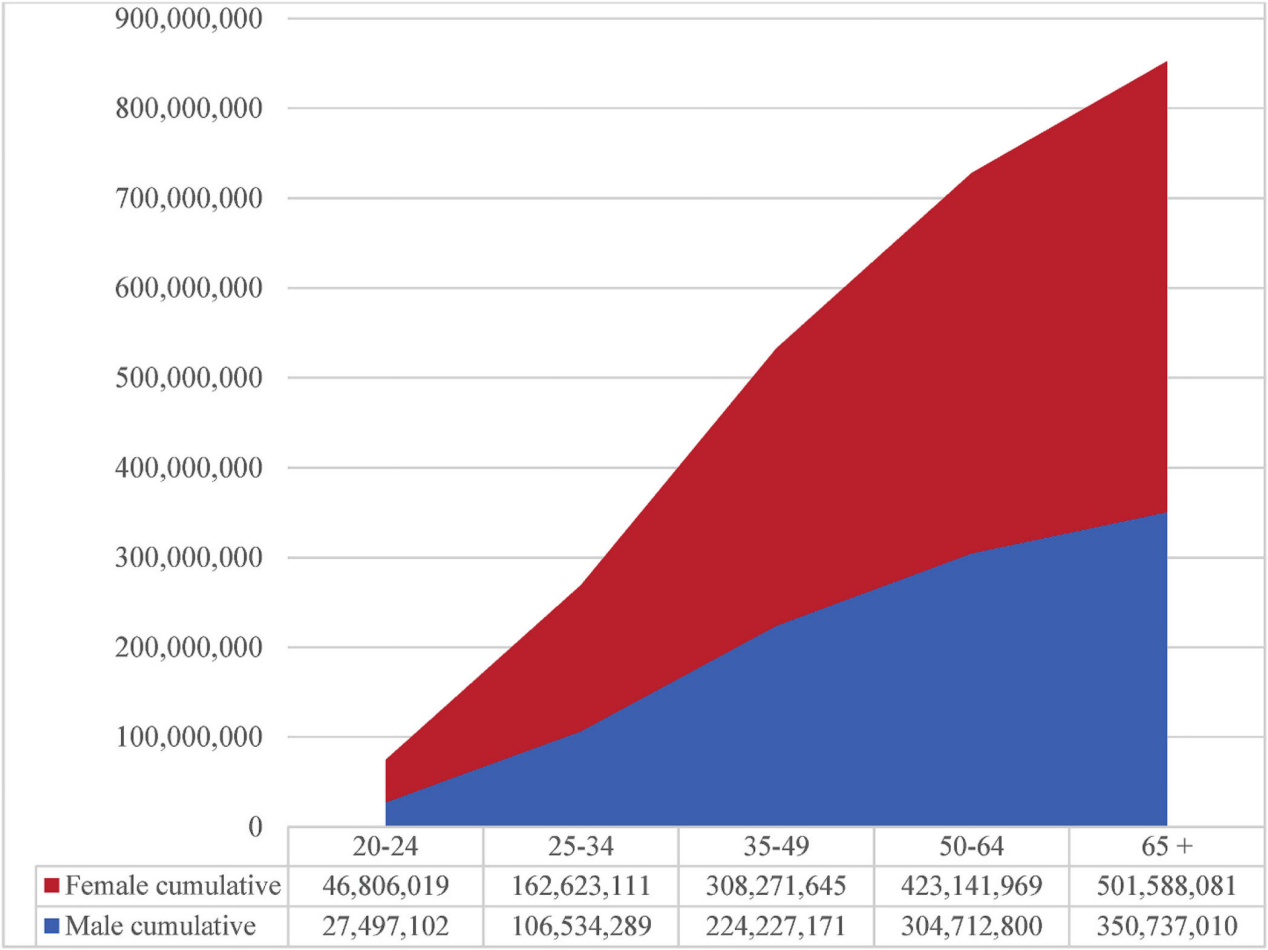
Cumulative number of adults with insomnia disorder by sex and age group This figure shows the cumulative number of adults with insomnia disorder, stratified by sex and age threshold (e.g., ≥20, ≥30, ≥40 years, etc.). Values represent modeled estimates based on sex- and age-specific prevalence rates applied to UN 2022 population data. This cumulative format illustrates the increasing burden of insomnia with advancing age.

The overall prevalence of clinically relevant insomnia was higher in females (18.9 %; 501,588,081 adults) than in males (13.4 %; 350,737,010 adults); this finding was also the case for severe insomnia, which was estimated to affect 9.9 % of females (261,863,183 individuals) and 5.9 % of males (153,104,758 individuals) (Fig. 3). A higher prevalence of insomnia in adult females versus males was evident across all age groups (Fig. 4). To complement the cumulative age-threshold data presented in Fig. 4, Supplementary Table S2 provides the estimated number of adults with insomnia disorder by sex and age.

### 3.4. Sensitivity analyses

Applying the prevalence of severe insomnia from Leger et al. [24] to all countries/territories without a local reference study resulted in an estimated global insomnia prevalence of 10.5 % (554,239,030 adults). Applying the prevalence of insomnia and severe insomnia from Leger et al. [24] to all countries resulted in global prevalence estimates of 18.5 % (973,603,071 adults) and 9.3 % (489,406,925 adults), respectively. The global prevalence of severe insomnia in adults was 7.9 % (414,967, 941 adults). The burden of severe insomnia for all countries is shown in a heat map in Fig. S3.

## 4. Discussion

To the best of our knowledge, this is the first study to estimate the global population prevalence of insomnia in adults based on aggregation of published data. By combining available prevalence data with UN population statistics, we estimate that over 850 million adults aged ≥20 years worldwide (or 16.2 % of the global population) experience clinically relevant insomnia, and that almost half of these individuals (415 million) have severe insomnia. These results indicate that chronic insomnia is common worldwide, with a population prevalence that is similar to the 2022 prevalence of obesity in adults aged ≥18 years [41] and approaching the estimated global prevalence of obstructive sleep apnea in adults aged 30–69 years [42].

The current analysis confirmed the existing understanding that there is a higher prevalence of insomnia in females compared with males [5, 43–45]. For example, a US analysis of individuals who participated in seven consecutive National Health and Nutrition Examination Survey cycles from 2005 to 2018 included a similar adult population as the reference study for the current analysis (age ≥20 years) and also found that insomnia was more prevalent in females than in males [45]. Our findings are also consistent with the results of a meta-analysis of observational studies that found a significantly higher prevalence of insomnia in females versus males [44]. In contrast, our results pertaining to the prevalence of insomnia at different ages are somewhat inconsistent with prior literature. Similar to previous studies, we found that the prevalence of clinically relevant insomnia increased up to the age of 35–49 years for both males and females. However, surprisingly we found that the prevalence of insomnia declined slightly among adults aged ≥50 years. One possible explanation is our clearly defined and specific definition of insomnia. Existing data suggest that individuals of different ages who have insomnia might report different types of sleep difficulties, with difficulty falling asleep being more common in younger individuals and difficulty maintaining sleep being more problematic for older individuals [46]. Such granular assessment of specific insomnia symptoms was not possible in the current study, which focused on the overall prevalence of insomnia disorder.

Our findings suggest important future directions in terms of research and knowledge gaps, as well as public health. Most importantly, there is urgent need for much greater emphasis on sleep from a global health perspective. Not only is sleep a biological necessity and basic human right [47], in developed nations sleep is widely recognized as modifiable health behavior, akin to nutrition and physical activity [48]. Insufficient and disturbed sleep, and clinical sleep disorders such as chronic insomnia, can impact virtually all bodily functions and are robustly associated with a broad array of adverse medical and psychiatric outcomes, including premature death, as well as dramatically increased economic burden [9–14,17,49]. Despite this notion, our comprehensive literature search only identified population prevalence estimates of chronic insomnia, the most common sleep disorder seen in clinical practice, for 31 of 237 countries/territories worldwide. This finding is consistent with prior efforts highlighting the paucity of high-quality sleep health surveillance data from a global perspective [50]. As noted elsewhere [51], disadvantaged individuals worldwide experience particularly high rates of sleep problems, further highlighting the importance of sleep from a health equity perspective. Unsafe sleep environments, unstable housing, and food insecurity all contribute to disparities in sleep health. Related to this point, greater understanding of cultural aspects and perceptions of sleep is an important direction for future research, which will help to guide the development of culturally relevant and tailored self-report measures of insomnia and other aspects of sleep. Such efforts should include assessment of both acute as well as chronic insomnia using an accepted and standardized nosology. Finally, in terms of public health, improving awareness of sleep health will require sustained effort from diverse stakeholders including, at a minimum, scientists, clinicians, educators, public leaders, and policy makers. Education, surveillance, and inclusion of sleep in public health policy-making will be vital next steps.

Our study has several strengths. Most importantly, our analysis represents the first effort to quantify the worldwide prevalence of chronic insomnia in adults. Using an established, literature-based methodology [42], we aggregated published, peer-reviewed findings regarding insomnia prevalence and applied these to global population data from the UN. Recognizing the heterogeneity of our data sources, we also employed rigorous, conservative research approaches throughout, performing multiple sensitivity analyses, and reporting the likely prevalence of both insomnia disorder and severe insomnia based on clear definitions from the reference studies. Thus, we employed innovative and scientifically rigorous methods to address a timely and important research question with global impact.

At the same time, the results from this novel study need to be interpreted in the context of several limitations. The main limitation is the small number of country-specific insomnia prevalence estimates that were used to estimate the global prevalence of chronic insomnia. To overcome this limitation, we applied data from the highest quality published study [24] to countries and territories without local insomnia prevalence estimates. Even so, our global prevalence figures are driven largely by data from a single robust study [24], which may not fully capture regional variations in insomnia prevalence driven by race, ethnicity, cultural and social determinants of sleep health, healthcare access, and societal stressors. This limitation is especially important in regions where sleep health is under-recognized, where stigma limits reporting, or where populations are exposed to structural inequities or social instability that may increase the burden of insomnia. As a result, our modeling approach may over- or underestimate the true burden in specific settings. A related limitation is our limited ability to assess perceptions of sleep or the cultural and social determinants of sleep health that differ between geographic regions and across sociodemographic groups [52–54]. Although the limiting available data in the literature does influence our results, we hope that our efforts help to encourage further research into this important problem.

Also, because we performed a literature-based analysis, the current findings are subject to the same limitations as the primary studies. For example, many of the available insomnia studies were performed in high-income regions and did not necessarily perform systematic population-level assessments. Another important limitation is the heterogeneity of insomnia assessment in the included studies. To maximize the number of prevalence estimates available for our analysis, we included studies performed within different timeframes, during which the diagnostic criteria for insomnia changed. Finally, were also unable to assess the impact of natural disasters or the COVID-19 pandemic, which have been linked with changes in rates of insomnia in some countries [55,56]. Future surveillance efforts should incorporate culturally relevant, tailored measures of sleep including insomnia symptoms.

## 5. Conclusions

This analysis highlights the high worldwide prevalence of insomnia in adults, and documents variations by country/territory. There is a major need for more robust studies investigating the prevalence of insomnia, especially in places where there is a current lack of published data. The high global prevalence of insomnia disorder identified in this analysis, the documented associations between insomnia and a variety of physical and mental health conditions, and the economic burden of insomnia highlight the worldwide need for a robust approach to sleep health.

## Supplementary Material

sup

## Figures and Tables

**Table 1:** Studies reporting country-specific data on insomnia prevalence.

Country	Year	Sample, n	Clinical definition	Female	Age range, years	Insomnia disorder		Severe insomnia	
						Male	Female	Male	Female

Belgium [5]	1999	1618	ICSD-2	–	≥15	11.0 %		5.0 %	5.8 %

Brazil [40]	2013	1101	DSM-IV	53.7 %	20–29	12.2 %		5.5 %	6.5 %
					30–39	21.7 %		9.8 %	11.5 %
					40–49	13.6 %		6.1 %	7.2 %
					50–59	16.0 %		7.2 %	8.5 %
					60–80	9.5 %		4.3 %	5.1 %
								
Canada [4]	2006	2001	DSM-IV	51.0 %	20–29	7.3 %		3.3 %	3.9 %
					30–39	9.2 %		4.1 %	4.9 %
					40–49	10.7 %		4.8 %	5.7 %
					50–59	11.4 %		5.1 %	6.0 %
					60–69	9.5 %		4.3 %	5.0 %
					≥70	8.1 %		3.6 %	4.3 %
								
China [28]	2017	115,988	Multiple	–	≥15	15.0 %		6.8 %	8.0 %

France [24]	2000	12,778	DSM-IV	53.0 %	20–24	7.4 %	17.4 %	2.0 %	5.2 %
					25–34	12.6 %	22.9 %	4.7 %	10.3 %
					35–49	16.3 %	22.8 %	6.6 %	12.1 %
					50–64	14.6 %	24.5 %	7.6 %	14.8 %
					≥65	15.5 %	25.1 %	8.6 %	16.2 %
									
Germany [30]	2001	1913	DSM-IV	53.0 %	18–24	1.0 %		0.5 %	0.5 %
					25–34	3.0 %		1.4 %	1.6 %
					35–44	4.0 %		1.8 %	2.1 %
					45–54	5.0 %		2.3 %	2.7 %
					55–64	7.0 %		3.2 %	3.7 %
					≥65	4.0 %		1.8 %	2.1 %
								
Italy [35]	2002	3970	DSM-IV	52.0 %	≥15	7.0 %		3.2 %	3.7 %

Japan [39]	2016	2614	DSM-IV	54.5 %	20–29	2.9 %	3.4 %	1.3 %	1.8 %
					30–39	2.8 %	1.8 %	1.3 %	1.0 %
					40–49	2.8 %	2.9 %	1.3 %	1.5 %
					50–59	4.7 %	3.2 %	2.1 %	1.7 %
					60–69	3.1 %	4.3 %	1.4 %	2.3 %
					≥70	3.1 %	9.1 %	1.4 %	4.8 %
									
Malaysia [28]	2008	1611	DSM-IV	47.1 %	30 to 70	9.6 %		4.3 %	5.1 %

Norway [37]	2020	21,083	ICSD-3	51.9 %	20–39	16.3 %	18.5 %	7.3 %	9.8 %
					40–44	16.3 %	18.5 %	7.3 %	9.8 %
					45–49	16.6 %	24.0 %	7.5 %	12.7 %
					50–54	17.1 %	26.8 %	7.7 %	14.2 %
					55–59	16.4 %	27.3 %	7.4 %	14.5 %
					60–64	14.6 %	24.7 %	6.6 %	13.1 %
					65–69	11.2 %	23.9 %	5.0 %	12.7 %
					70–74	12.7 %	27.3 %	5.7 %	14.5 %
					75–79	13.1 %	25.9 %	5.9 %	13.7 %
					≥80	13.9 %	28.1 %	6.3 %	14.9 %
									
Qatar [33]	2021	1611	DSM-V	53.2 %	≥18	3.0 %		1.4 %	1.6 %

South Korea [34]	2002	3719	DSM-IV	50.5 %	≥15	4.7 %	5.1 %	2.1 %	2.7 %

Spain [29]	2023	2115	ICSD-3	52.2 %	20–34	11.1 %		5.0 %	5.9 %
					35–54	11.5 %		5.2 %	6.1 %
					≥55	17.9 %		8.1 %	9.5 %
								
Sweden [5]	1999	1996	ICSD-2	–	≥15	13 %		5.9 %	6.9 %

Taiwan [31]	2008	9298	ISAI	50.4 %	≥18	9.3 %		4.2 %	4.9 %

The Netherlands [32]	2017	2089	ICSD-2	–	20–24	3.6 %	10.9 %	1.6 %	5.8 %
					25–34	6.5 %	6.4 %	2.9 %	3.4 %
					35–44	8.9 %	13.3 %	4.0 %	7.0 %
					45–54	8.5 %	7.3 %	3.8 %	3.9 %
					55–70	4.8 %	9.4 %	2.2 %	5.0 %
									
Turkey [27]	2015	4758	DSM-IV	51.7 %	20–24	9.8 %		4.4 %	5.2 %
					25–44	11.7 %		5.3 %	6.2 %
					45–64	13.8 %		6.2 %	7.3 %
					≥65	13.9 %		6.3 %	7.4 %
								
UK [5]	2001	4972	ICSD-2	–	≥15	9.1 %		4.1 %	4.8 %

USA [36]	2011	10,094	RDC	–	≥18	14.7 %		6.6 %	7.8 %

Multiple countries^a^ [26]	2021	57,298	DSM-IV	53 %	20–29	11.1 %		5.0 %	5.9 %
					30–39	11.7 %		5.3 %	6.2 %
					40–49	12.4 %		5.6 %	6.6 %
					50–59	11.9 %		5.4 %	6.3 %
					≥60	9.9 %		4.5 %	5.2 %

DSM-IV, Diagnostic and Statistical Manual of Mental Disorders Fourth Edition; DSM-V, Diagnostic and Statistical Manual of Mental Disorders Fifth Edition; ICSD-2, International Classification of Sleep Disorders Second Edition; ICSD-3, International Classification of Sleep Disorders Third Edition; ISAI, Insomnia Self-Assessment Inventory; RDC, research diagnostic criteria.

a Algeria, Andorra, French Polynesia, Greece, Guadeloupe, Mauritania, Mayotte, Morocco, New Caledonia, Seychelles, Tonga, Tunisia. Note: While 17 studies were included in the analysis, one study (Ohayon et al., *Sleep Med Rev*, 2002) reported data for three countries (Belgium, Sweden, and the UK), resulting in 19 country-level entries.

## Practice points.

1. More than 16 % of the global population is estimated to have insomnia.
2. The prevalence of insomnia is higher in females than in males.
3. There is an urgent need for greater emphasis on sleep from a public health perspective.

## Research agenda.

1. Directly determine the prevalence of insomnia and severe insomnia in the majority of countries/regions worldwide.
2. Obtain a greater understanding of cultural aspects and perceptions of sleep.
3. Improve awareness of sleep health among scientists, clinicians, educators, public leaders and policy makers.

## Appendix A. Supplementary data

Supplementary data to this article can be found online at https://doi.org/10.1016/j.smrv.2025.102121.

## Abbreviations

CI: confidence interval
DSM: Diagnostic and Statistical Manual of Mental Disorder
ICSD: International Classification of Sleep Disorders
UN: United Nations
