# Supplementary material for: Estimation of the global prevalence and burden of insomnia: a systematic literature review-based analysis
Source: Sleep Med Rev. Author manuscript; Available in PMC 2025 Dec 4. (PMC12676268; doi:10.1016/j.smrv.2025.102121)
Supplement: sup [file NIHMS2096202-supplement-sup.pdf]

Supplementary Data

Benjafield AV, et al. Estimation of the global prevalence and burden of insomnia: a literature-based analysis

Figure S1. The ten countries with the highest prevalence of insomnia disorder in adults

Estimates are based on modeled data applying age- and sex-stratified insomnia prevalence rates to United Nations 2022 population data. For countries without a country-specific prevalence study identified in our systematic review, data were modeled using prevalence estimates from Leger et al. (*J Sleep Res* 2000). See methods for additional detail.

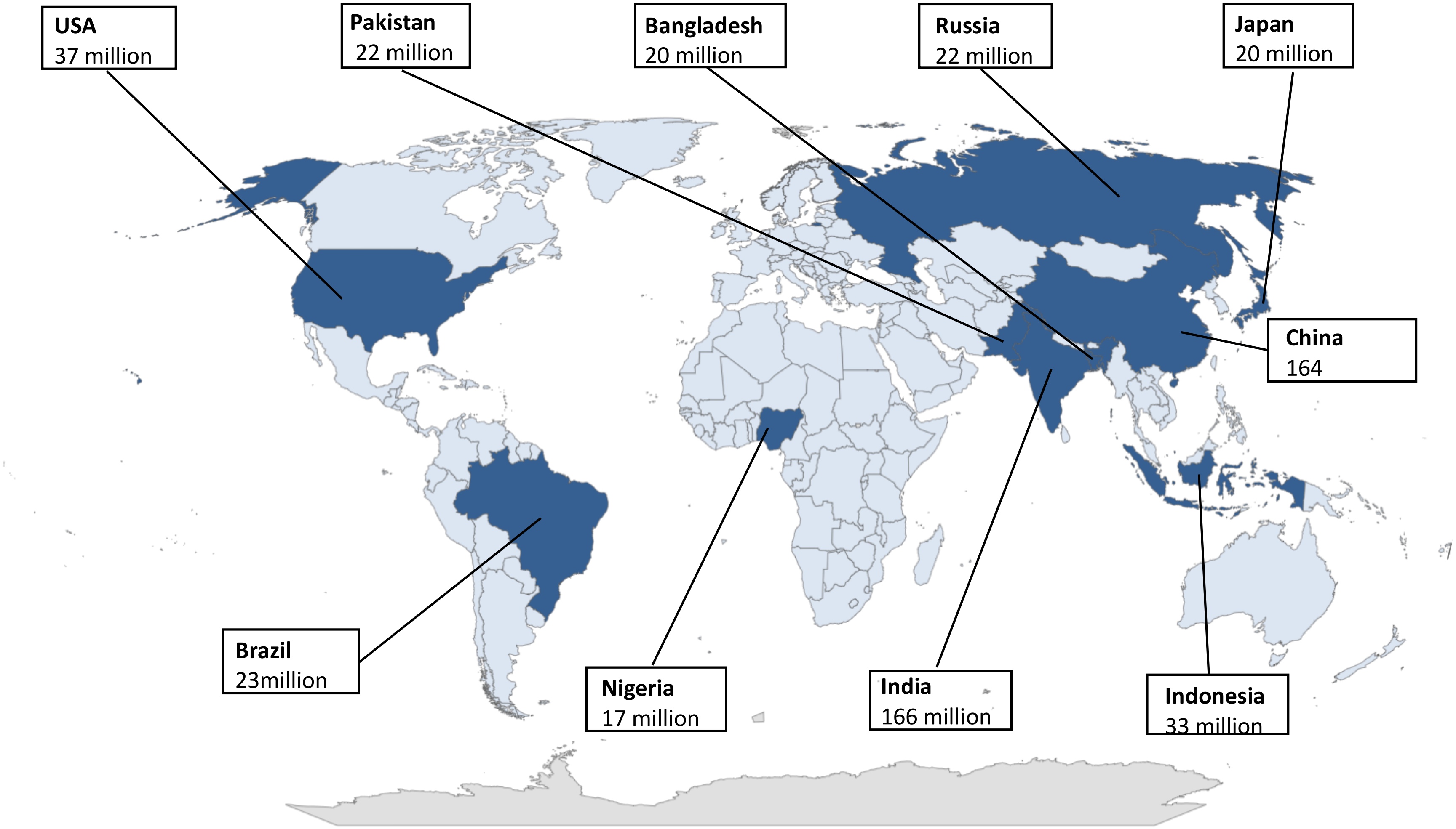
USA
37 million
Pakistan
22 million
Bangladesh
20 million
Russia
22 million
Japan
20 million
China
164
Brazil
23million
Nigeria
17 million
India
166 million
Indonesia
33 million

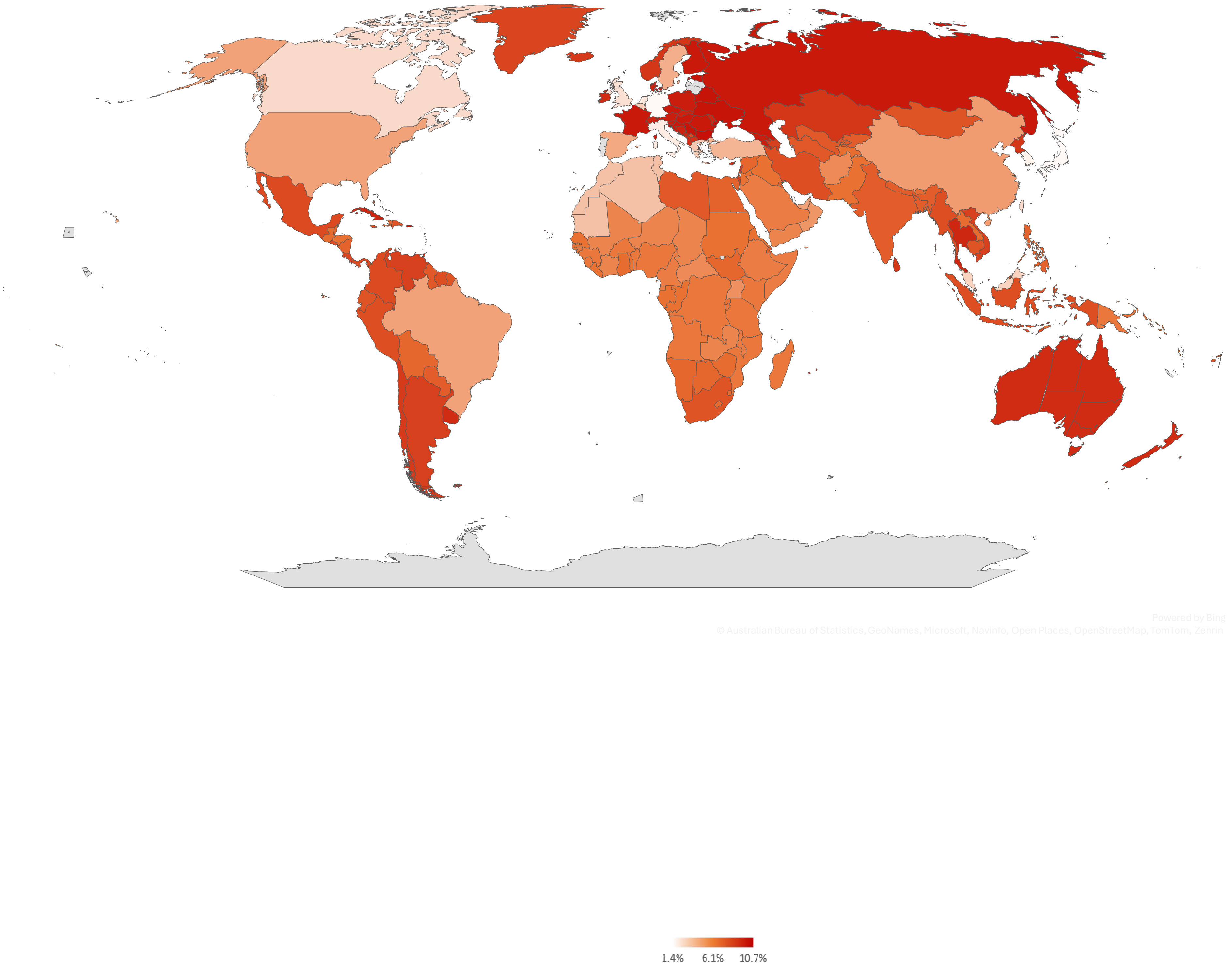
Powered by Bing
© Australian Bureau of Statistics, GeoNames, Microsoft, Navinfo, Open Places, OpenStreetMap, TomTom, Zenrin
1.4%
6.1%
10.7%

Table S1. Critical appraisal results for included studies using the Newcastle - Ottawa quality assessment Scale (NOS): (Adapted for cross-sectional studies)

Reference<br>First Author, year	Selection<br>(max. 5 stars)	Comparability<br>(max. 2 stars)	Outcome<br>(max. 3 stars)	Overall score<br>(max. 10)
Aernout et al, 2021[26]<br>(Multiple Countries)	★★★★	★	★★	7
Benbir et al, 2015[27]<br>(Turkey)	★★★★	★	★★	7
Cao et al, 2017[28]<br>(China)	★★★★		★★	6
Castro et al, 2013[40]<br>(Brazil)	★★★★	★★	★	7
de Entrambasaguas et al, 2023[29]<br>(Spain)	★★★★	★	★★	7
Hajak et al, 2001[30]<br>(Germany)	★★★★	★	★★	7
Itani et al, 2016[39]<br>(Japan)	★★★★	★★	★★	8
Kao et al, 2008[31]<br>(Taiwan)	★★★★		★★	6
Kerkhof et al, 2017[32]<br>(Netherlands)	★★★★	★★	★★	8
Khaled et al, 2021[33]<br>(Qatar)	★★★★		★★	6
Leger et al, 2000[24]<br>(France)	★★★★	★★	★★	8
Morin et al, 2006[4]<br>(Canada)	★★★★	★	★★	7
Ohayon et al, 2002[34]<br>(South Korea)	★★★★	★★	★★	7
Ohayon et al, 2002[35]<br>(Italy)	★★★★	★★	★★	8
Ohayon et al, 2002[5]<br>(UK, BE, Sweden)	★★★★		★★	6
Roth et al, 2011[36]<br>(USA)	★★★★		★★	6
Siversten et al, 2021[37]<br>(Norway)	★★★★	★★	★★	8
Zailinawati et al, 2008[38]<br>(Malaysia)	★★★★	★	★★	7

Table S2. Estimated number of adults with insomnia disorder by sex and age group

Age Group (years)	Males (n)	Females (n)	Total (n)
20-24	27,497,102	46,806,019	74,303,121
25-34	79,037,187	115,817,092	194,854,279
35-49	117,692,882	145,648,533	263,341,415
50-64	80,485,630	114,870,324	195,355,954
65 +	46,024,210	78,446,112	124,470,325
Total	350,737,010	501,588,081	852,325,091

Note: Values represent modeled estimates based on age- and sex-specific prevalence data applied to 2022 UN population data.

Table S3. Number of people with, and prevalence of, insomnia disorder and severe insomnia in adults by country

Country	Population aged ≥20 years	Insomnia disorder		Severe insomnia	
		Number	Prevalence rate	Number	Prevalence rate
Afghanistan	17,991,959	3,137,634	17.4%	1,422,833	7.9%
Albania	2,203,557	411,873	18.7%	211,110	9.6%
Algeria	27,482,119	3,152,168	11.5%	1,542,366	5.6%
American Samoa	28,927	5,353	18.5%	2,641	9.1%
Andorra	64,600	7,332	11.4%	3,587	5.6%
Angola	15,329,335	2,729,693	17.8%	1,261,795	8.2%
Anguilla	12,140	2,295	18.9%	1,155	9.5%
Antigua and Barbuda	69,240	13,043	18.8%	6,580	9.5%
Argentina	31,222,887	5,811,415	18.6%	2,908,190	9.3%
Armenia	2,059,836	399,020	19.4%	205,406	10.0%
Aruba	81,613	15,731	19.3%	8,264	10.1%
Australia	19,656,826	3,701,133	18.8%	1,899,040	9.7%
Austria	7,204,867	1,373,662	19.1%	720,525	10.0%
Azerbaijan	7,160,770	1,339,232	18.7%	663,314	9.3%
Bahamas	294,740	55,067	18.7%	27,363	9.3%
Bahrain	1,087,091	186,897	17.2%	86,113	7.9%
Bangladesh	107,801,904	19,568,389	18.2%	9,364,901	8.7%
Barbados	214,754	40,893	19.0%	21,194	9.9%
Belarus	7,490,722	1,458,091	19.5%	765,338	10.2%
Belgium	9,026,146	992,876	11.0%	487,405	5.4%
Belize	248,019	44,571	18.0%	21,067	8.5%
Benin	6,105,390	1,083,326	17.7%	502,129	8.2%
Bermuda	51,270	9,897	19.3%	5,257	10.3%
Bhutan	528,385	93,843	17.8%	44,218	8.4%
Bolivia	7,147,586	1,287,458	18.0%	609,918	8.5%
Bonaire, Sint Eustatius and Saba	20,780	3,906	18.8%	2,004	9.6%
Bosnia and Herzegovina	2,615,284	497,539	19.0%	259,687	9.9%
Botswana	1,494,420	271,025	18.1%	127,495	8.5%
Brazil	154,392,012	22,683,482	14.7%	11,162,977	7.2%
British Virgin Islands	24,308	4,598	18.9%	2,320	9.5%
Brunei Darussalam	312,227	57,027	18.3%	27,691	8.9%
Bulgaria	5,607,049	1,083,992	19.3%	575,690	10.3%
Burkina Faso	9,953,405	1,764,262	17.7%	811,595	8.2%
Burundi	5,417,022	965,063	17.8%	443,726	8.2%
Cambodia	10,267,757	1,886,571	18.4%	32,658	8.6%
Cameroon	12,765,236	2,260,807	17.7%	914,441	8.9%
Canada	30,101,011	2,819,893	9.4%	1,038,519	8.1%
Cape Verde	378,822	68,659	18.1%	1,383,247	4.6%
Cayman Islands	53,807	10,067	18.7%	5,002	9.3%
Central African Republic	2,159,336	374,794	17.4%	170,667	7.9%
Chad	7,150,000	1,254,829	17.6%	571,812	8.0%
Chile	14,667,636	2,737,499	18.7%	1,377,832	9.4%
China	1,094,457,303	164,168,595	15.0%	80,406,990	7.3%
China, Hong Kong	6,315,516	1,228,467	19.5%	650,188	10.3%
China, Macao SAR	559,725	106,543	19.0%	54,039	9.7%
Colombia	36,289,397	6,697,185	18.5%	3,295,647	9.1%
Comoros	427,150	76,296	17.9%	35,818	8.4%
Congo	2,826,199	505,958	17.9%	235,938	8.3%
Cook Islands	11,663	2,207	18.9%	1,126	9.7%
Costa Rica	3,725,524	690,083	18.5%	343,454	9.2%

Country	Population aged ≥20 years	Insomnia disorder		Severe insomnia	
		Number	Prevalence rate	Number	Prevalence rate
Cote d'Ivoire	12,922,101	2,267,291	17.5%	1,033,747	8.0%
Croatia	3,290,320	631,183	19.2%	333,880	10.1%
Cuba	8,843,611	1,671,714	18.9%	866,587	9.8%
Curacao	143,711	27,141	18.9%	13,974	9.7%
Cyprus	982,320	184,840	18.8%	93,957	9.6%
Czech Republic	8,337,489	1,600,287	19.2%	840,184	10.1%
Democratic People's Republic of Korea	19,322,441	3,618,150	18.7%	1,834,704	9.5%
Democratic Republic of the Congo	41,351,201	7,320,016	17.7%	3,381,992	8.2%
Denmark	4,564,613	863,561	18.9%	451,199	9.9%
Djibouti	651,145	117,611	18.1%	55,681	8.6%
Dominica	52,384	9,642	18.4%	4,764	9.1%
Dominican Republic	7,100,849	1,295,310	18.2%	628,930	8.9%
Ecuador	11,585,400	2,115,145	18.3%	1,025,734	8.9%
Egypt	63,504,695	11,497,782	18.1%	5,486,436	8.6%
El Salvador	4,102,432	754,972	18.4%	368,084	9.0%
Equatorial Guinea	863,426	151,891	17.6%	70,192	8.1%
Eritrea	1,746,006	311,051	17.8%	145,768	8.3%
Estonia	1,043,990	202,280	19.4%	106,858	10.2%
Ethiopia	58,752,253	10,355,848	17.6%	4,763,325	8.1%
Falkland Islands (Malvinas)	2,879	547	19.0%	278	9.7%
Faroe Islands	38,248	7,145	18.7%	3,709	9.7%
Fiji	577,733	105,555	18.3%	51,069	8.8%
Finland	4,381,412	836,155	19.1%	441,495	10.1%
France	49,411,234	9,485,293	19.2%	5,013,459	10.1%
French Guiana	173,086	31,586	18.2%	15,328	8.9%
French Polynesia	215,222	24,478	11.4%	11,988	5.6%
Gabon	1,273,748	227,461	17.9%	106,308	8.3%
Gambia	1,199,677	212,421	17.7%	97,788	8.2%
Georgia	2,755,359	529,977	19.2%	274,935	10.0%
Germany	67,975,547	2,973,037	4.4%	1,459,803	2.1%
Ghana	17,255,952	3,100,125	18.0%	1,451,745	8.4%
Gibraltar	25,191	4,776	19.0%	2,508	10.0%
Greece	8,428,609	939,029	11.1%	461,278	5.5%
Greenland	40,986	7,504	18.3%	3,757	9.2%
Grenada	85,906	15,861	18.5%	7,847	9.1%
Guadeloupe	294,600	32,702	11.1%	16,199	5.5%
Guam	112,045	20,721	18.5%	10,489	9.4%
Guatemala	9,924,531	1,777,127	17.9%	832,529	8.4%
Guernsey	48,932	9,205	18.8%	4,740	9.7%
Guinea	6,405,472	1,136,035	17.7%	525,588	8.2%
Guinea-Bissau	999,263	178,328	17.8%	82,316	8.2%
Guyana	500,259	91,406	18.3%	44,241	8.8%
Haiti	6,600,688	1,188,601	18.0%	560,203	8.5%
Honduras	6,064,497	1,082,841	17.9%	505,806	8.3%
Hungary	7,806,860	1,503,928	19.3%	790,185	10.1%
Iceland	278,956	51,807	18.6%	26,266	9.4%
India	917,582,853	165,844,592	18.1%	79,945,837	8.7%
Indonesia	181,790,579	33,393,645	18.4%	16,331,635	9.0%
Iran	61,291,073	11,345,799	18.5%	5,546,717	9.0%
Iraq	22,442,072	4,008,486	17.9%	1,869,239	8.3%
Ireland	3,667,357	692,392	18.9%	355,127	9.7%
Isle of Man	67,305	12,881	19.1%	6,845	10.2%
Israel	5,697,178	1,059,282	18.6%	532,005	9.3%
Italy	48,898,345	3,422,884	7.0%	1,682,248	3.4%
Jamaica	2,024,323	371,887	18.4%	181,223	9.0%

Country	Population aged ≥20 years	Insomnia disorder		Severe insomnia	
		Number	Prevalence rate	Number	Prevalence rate
Japan	104,338,502	4,340,282	4.2%	2,173,401	2.1%
Jersey	87,685	16,552	18.9%	8,513	9.7%
Jordan	6,449,301	1,144,372	17.7%	535,607	8.3%
Kazakhstan	12,228,189	2,311,577	18.9%	1,157,573	9.5%
Kenya	26,672,796	4,742,879	17.8%	2,184,078	8.2%
Kiribati	71,079	12,914	18.2%	6,113	8.6%
Kosovo	1,142,731	210,623	18.4%	104,555	9.1%
Kuwait	3,123,944	557,917	17.9%	268,610	8.6%
Kyrgyzstan	3,750,792	685,400	18.3%	328,095	8.7%
Lao People's Democratic Republic	4,399,332	788,003	17.9%	370,360	8.4%
Latvia	1,489,926	291,376	19.6%	155,657	10.4%
Lebanon	3,554,921	665,138	18.7%	334,017	9.4%
Lesotho	1,274,823	228,780	17.9%	107,006	8.4%
Liberia	2,483,358	442,972	17.8%	206,400	8.3%
Libya	4,160,466	757,959	18.2%	364,200	8.8%
Liechtenstein	31,407	5,987	19.1%	3,144	10.0%
Lithuania	2,236,909	433,830	19.4%	230,534	10.3%
Luxembourg	504,241	94,738	18.8%	48,392	9.6%
Madagascar	14,437,139	2,558,866	17.7%	1,185,821	8.2%
Malawi	8,962,190	1,594,437	17.8%	728,033	8.1%
Malaysia	23,166,241	2,223,959	9.6%	1,088,030	4.7%
Maldives	377,376	65,706	17.4%	30,454	8.1%
Mali	9,098,566	1,594,421	17.5%	726,850	8.0%
Malta	435,882	81,273	18.6%	41,482	9.5%
Marshall Islands	24,634	4,495	18.2%	2,163	8.8%
Martinique	285,075	55,381	19.4%	29,860	10.5%
Mauritania	2,173,423	248,513	11.4%	122,292	5.6%
Mauritius	988,473	185,162	18.7%	93,820	9.5%
Mayotte	143,992	16,567	11.5%	8,184	5.7%
Mexico	84,216,020	15,583,496	18.5%	7,655,613	9.1%
Micronesia	66,663	11,956	17.9%	5,724	8.6%
Monaco	30,533	5,932	19.4%	3,279	10.7%
Mongolia	2,039,423	376,196	18.4%	181,917	8.9%
Montenegro	475,453	90,361	19.0%	46,762	9.8%
Montserrat	3,529	650	18.4%	335	9.5%
Morocco	24,115,929	2,750,791	11.4%	1,347,943	5.6%
Mozambique	14,567,237	2,586,473	17.8%	1,189,636	8.2%
Myanmar	35,811,792	6,585,083	18.4%	3,211,126	9.0%
Nambia	1,376,078	249,044	18.1%	117,135	8.5%
Nauru	6,524	1,167	17.9%	543	8.3%
Nepal	17,985,085	3,280,346	18.2%	1,563,658	8.7%
Netherlands	13,766,356	1,072,115	7.8%	534,193	3.9%
New Caledonia	201,639	22,913	11.4%	11,241	5.6%
New Zealand	3,841,462	722,418	18.8%	370,875	9.7%
Nicaragua	4,140,141	750,430	18.1%	356,269	8.6%
Niger	10,178,452	1,778,782	17.5%	814,136	8.0%
Nigeria	98,099,225	17,340,606	17.7%	8,018,286	8.2%
Niue	1,296	251	19.4%	131	10.1%
Northern Mariana Islands	35,834	6,562	18.3%	3,355	9.4%
Norway	4,169,026	797,683	19.1%	396,874	9.5%
Oman	3,054,060	513,940	16.8%	230,526	7.5%
Pakistan	121,141,851	21,568,534	17.8%	10,112,089	8.3%
Palau	13,027	2,408	18.5%	1,210	9.3%
Panama	2,844,527	523,894	18.4%	257,993	9.1%
Papua New Guinea	5,496,755	974,459	17.7%	453,217	8.2%
Paraguay	4,159,212	753,454	18.1%	361,051	8.7%

Country	Population aged ≥20 years	Insomnia disorder		Severe insomnia	
		Number	Prevalence rate	Number	Prevalence rate
Peru	21,954,857	4,040,406	18.4%	1,975,469	9.0%
Philippines	68,229,909	12,318,058	18.1%	5,886,608	8.6%
Poland	30,612,513	5,878,884	19.2%	3,067,747	10.0%
Portugal	8,382,311	1,624,091	19.4%	864,114	10.3%
Puerto Rico	2,626,201	506,261	19.3%	268,244	10.2%
Qatar	2,187,047	65,611	3.0%	30,692	1.4%
Republic of Korea	43,297,882	2,122,235	4.9%	1,043,984	2.4%
Republic of Moldova	2,288,248	437,803	19.1%	225,040	9.8%
Réunion	665,870	125,716	18.9%	64,590	9.7%
Romania	15,202,741	2,919,816	19.2%	1,531,020	10.1%
Russian Federation	112,048,669	21,732,695	19.4%	11,327,327	10.1%
Rwanda	6,731,834	1,208,124	17.9%	560,760	8.3%
Saint Barthélemy	9,013	1,666	18.5%	832	9.2%
Saint Helena	4,452	861	19.3%	469	10.5%
Saint Kitts and Nevis	35,032	6,553	18.7%	3,278	9.4%
Saint Lucia	133,790	24,762	18.5%	12,249	9.2%
Saint Martin	22,768	4,406	19.4%	2,291	10.1%
Saint Pierre and Miquelon	4,550	872	19.2%	460	10.1%
Saint Vincent and the Grenadines	73,807	13,604	18.4%	6,801	9.2%
Samoa	115,350	20,788	18.0%	9,958	8.6%
San Marino	27,629	5,321	19.3%	2,828	10.2%
Sao Tome and Principe	108,694	19,478	17.9%	9,147	8.4%
Saudi Arabia	23,850,931	4,170,701	17.5%	1,927,116	8.1%
Senegal	8,022,966	1,436,425	17.9%	667,625	8.3%
Serbia	5,881,783	1,135,051	19.3%	598,475	10.2%
Seychelles	74,813	8,556	11.4%	4,168	5.6%
Sierra Leone	4,180,816	740,431	17.7%	342,559	8.2%
Singapore	4,970,474	923,227	18.6%	471,267	9.5%
Sint Maarten	34,241	6,255	18.3%	3,231	9.4%
Slovakia	4,318,983	825,874	19.1%	428,504	9.9%
Slovenia	1,701,545	324,218	19.1%	170,514	10.0%
Solomon Islands	357,353	63,562	17.8%	29,674	8.3%
Somalia	7,152,264	1,256,944	17.6%	576,335	8.1%
South Africa	37,651,409	6,952,962	18.5%	3,365,193	8.9%
South Sudan	4,634,645	834,792	18.0%	394,909	8.5%
Spain	38,320,978	5,398,143	14.1%	2,654,235	6.9%
Sri Lanka	14,970,755	2,820,390	18.8%	1,427,467	9.5%
State of Palestine	2,587,711	460,548	17.8%	213,819	8.3%
Sudan	22,290,681	3,966,713	17.8%	1,838,998	8.3%
Suriname	395,624	72,609	18.4%	35,574	9.0%
Swaziland	651,903	116,955	17.9%	54,637	8.4%
Sweden	8,018,856	1,042,451	13.0%	510,797	6.4%
Switzerland	6,961,673	1,323,943	19.0%	691,343	9.9%
Syrian Arab Republic	11,599,752	2,077,464	17.9%	986,431	8.5%
Taiwan	19,693,269	1,831,474	9.3%	898,829	4.6%
Tajikstan	5,302,940	951,139	17.9%	445,930	8.4%
Macedonia	1,641,840	309,042	18.8%	158,073	9.6%
Thailand	56,141,839	10,646,892	19.0%	5,484,755	9.8%
Timor Leste	697,673	123,174	17.7%	57,589	8.3%
Togo	4,263,638	757,802	17.8%	352,075	8.3%
Tokelau	1,136	210	18.5%	104	9.2%
Tonga	58,099	6,616	11.4%	3,253	5.6%
Trinidad and Tobago	1,133,532	213,072	18.8%	107,346	9.5%
Tunisia	8,365,946	953,584	11.4%	468,384	5.6%
Turkey	58,559,045	7,264,964	12.4%	3,563,158	6.1%
Turkmenistan	3,866,979	708,257	18.3%	341,349	8.8%

Country	Population aged ≥20 years	Insomnia disorder		Severe insomnia	
		Number	Prevalence rate	Number	Prevalence rate
Turks and Caicos Islands	35,066	6,532	18.6%	3,261	9.3%
Tuvalu	6,715	1,216	18.1%	591	8.8%
Uganda	19,667,990	3,421,401	17.4%	1,530,688	7.8%
Ukraine	34,861,554	6,782,404	19.5%	3,557,523	10.2%
United Arab Emirates	7,640,130	1,247,604	16.3%	550,311	7.2%
United Kingdom	51,646,928	4,699,870	9.1%	2,307,329	4.5%
United Republic of Tanzania	28,879,886	5,151,994	17.8%	2,382,188	8.2%
United States of America	253,474,047	37,260,685	14.7%	18,287,327	7.2%
United States Virgin Islands	75,468	14,697	19.5%	7,848	10.4%
Uruguay	2,517,550	475,294	18.9%	243,427	9.7%
Uzbekistan	21,256,902	3,887,829	18.3%	1,868,508	8.8%
Vanuatu	162,305	29,179	18.0%	13,648	8.4%
Venezuela	17,660,546	3,281,203	18.6%	1,635,339	9.3%
Viet Nam	68,496,241	12,799,535	18.7%	6,351,280	9.3%
Wallis and Futuna Islands	7,909	1,513	19.1%	782	9.9%
Western Sahara	385,936	68,801	17.8%	32,769	8.5%
Yemen	16,271,499	2,862,803	17.6%	1,302,727	8.0%
Zambia	8,892,423	1,569,948	17.7%	711,525	8.0%
Zimbabwe	7,666,058	1,388,661	18.1%	643,890	8.4%
